# Enhanced Inflammatory Activity of Endometriotic Lesions from the Rectovaginal Septum

**DOI:** 10.1155/2013/450950

**Published:** 2013-12-28

**Authors:** Dominic Bertschi, Brett D. McKinnon, Jakob Evers, Nick A. Bersinger, Michael D. Mueller

**Affiliations:** ^1^Department of Obstetrics and Gynecology, Inselspital, Berne University Hospital, 3010 Berne, Switzerland; ^2^Department of Clinical Research, University of Berne, 3010 Berne, Switzerland

## Abstract

Endometriosis is characterised by the growth of ectopic lesions at multiple locations outside the uterine cavity and may be considered a collection of distinct but related conditions. The exact aetiology of endometriosis is still not clear although a role for inflammation is increasingly accepted. We therefore investigated the inflammatory activity of eutopic tissue and that of the matching ectopic lesions from different locations by measuring the genetic expression of inflammatory chemokines and cytokines. The gene expression in matching eutopic and ectopic tissue was compared, as was the gene expression in lesions from different locations. A significantly higher mRNA expression of the chemokines ENA-78 and RANTES and the cytokines IL-6 and TNF**α** was observed in endometriotic lesions of the rectovaginal septum (RVS) compared to that of matching eutopic tissue. Comparisons across lesion locations showed a significantly higher expression of IL-6 and TNF**α** in the RVS compared to lesions from either the ovaries or the peritoneum. These results show that the production of some inflammatory chemokines and cytokines is significantly increased in the ectopic endometrial tissue compared to matching eutopic tissue. Furthermore, IL-6 and TNF**α** are produced in significantly higher quantities in RVS lesions compared to other lesions.

## 1. Introduction

Endometriosis is defined as the presence of endometrial tissue outside the uterine cavity. The most common symptoms leading to a diagnosis are dysmenorrhoea, pelvic pain, and reduced fertility [1]. It is a very prevalent disease affecting up to 10% of the reproductive-aged female population [2].

The precise aetiology of endometriosis is not yet clear. Currently, the most widely accepted theory is the implantation theory: retrograde menstruation can result in viable endometrial cells and fragments entering the peritoneal cavity [3] and once attached [4], they promote a chronic pelvic inflammatory response [5]. Retrograde menstruation however cannot explain all cases, as endometriotic lesions have been identified in diverse locations such as the brain [6]. It is broadly accepted however that most of the ectopic lesions can be separated into three main regions: (i) ovarian, (ii) rectovaginal septum (RVS), and (iii) peritoneum. Biochemical and pathological differences between the lesions found in these locations have led to suggestions that endometriosis may represent a collection of related but distinct conditions [7]. It is possible that the variability between these distinct but related lesions is what contributes to the enigmatic nature of the disease.

The contribution of inflammation to the progression of endometriosis is increasingly being recognised. Endometriotics lesions that are established at ectopic sites secrete chemokines which attract macrophages into the peritoneal cavity, further stimulating the inflammatory response and release of cytokines [8]. Significantly increased numbers of activated macrophages have been identified in the peritoneal fluid of women with endometriosis [9], as has an increased concentration of various chemokines and cytokines. Significantly elevated levels of epithelial neutrophil-activating peptide (ENA-78) [10], monocyte chemotactic protein (MCP-1) [11], interleukin (IL)-8 [12], tumor necrosis factor (TNF)-*α* [13], IL-6 [14, 15], and regulated on activation normal T cell expressed and secreted (RANTES) [12] have all been found in the peritoneal fluid of patients with endometriosis. Underlining the inflammatory nature of the condition is the fact that TNF*α* [16], ENA-78 [17], and IL-6 [18, 19] are also elevated in the serum of women with endometriosis. Less data is however available on the inflammatory response of the lesion itself and whether there is variability based on the type or lesion location. A difference in the production of specific cytokines may provide an insight into the inflammatory activity of lesions that grow in different locations.

In order to gain a better understanding of this complex disease and the differences that can occur between various lesions, this study investigated the production of several chemokines and cytokines in matching eutopic endometrial and ectopic endometriotic tissue and compared their gene expression levels in the three most common presentations of the disease.

## 2. Patients and Methods

### 2.1. Sample Collection and Patient Data

Informed consent was collected prior to surgery from all women included in the study. Laparoscopic surgery was performed for the investigation of pelvic pain or infertility, and any endometriotic lesions identified were removed and their location was noted. Where possible, an endometrial biopsy was also collected using a soft curette (Pipelle-de-cornier, Laboratoire CCD, France). All tissue collected during the surgery was stored in RNAlater (Invitrogen Life Technologies, Zug, Switzerland) at −80°C until further use. Exclusion criteria for the study included prior or current infections, liver dysfunction, or the use of hormonal treatments, including any hormonal contraceptive or gonadotropin releasing hormone analogues (GnRHa) within the past 3 months. All laparoscopies were performed in the proliferative phase of the menstrual cycle. Institutional review board approval was obtained from the ethical committee prior to the commencement of the study.

After the informed consent was obtained and exclusion criteria were satisfied, we collected eutopic endometrial biopsies from 17 patients. A single matching ectopic lesion was collected from 15 women, two lesions were collected from another, and three lesions in the final case, resulting in 20 ectopic lesions with matching eutopic samples. The primary indication for surgery was dysmenorrhea for ten of these women, pelvic pain for four women, and infertility for the remaining three. The average age of the patients was 32.94 ± 1.454, range 24–41, and the body mass index (BMI) was 23.39 ± 0.914, range 18.90–33.10.

For the further comparison of the mRNA expression across ectopic sites additional lesions were collected from another 23 patients to make a total of 40 patients. A single lesion was collected from 34 patients, two lesions were collected from five patients, and three lesions were collected from one patient, resulting in a total of 47 ectopic endometriotic lesions. In some cases the isolated mRNA was insufficient to determine the concentration of all genes of interest and as such *n* values are included with each mean and SEM. The primary indication for surgery was dysmenorrhea for 17 women, pelvic pain for another 14, and idiopathic infertility for the remaining nine. The average age was 35.58 ± 1.265, range 22–58, and the BMI was 23.79 ± 0.811, range 18.00–47.30. No significant difference in either age or BMI was observed in the three groups based on lesion location.

### 2.2. Determination of Gene Expression in Eutopic Endometrium and Ectopic Endometriotic Tissue

Approximately 30 mg of tissue from both the eutopic endometrial biopsies and ectopic endometriotic lesions was excised and homogenized in the FastPrep 120 tissue homogenizer (30 seconds at 4.0 m/sec) in cell lysis buffer (Qiagen, Düsseldorf, Germany). RNA isolation was performed with the RNAeasy minikit (Qiagen) and after isolation the TurboDNase kit (Ambion, Life Technologies, Zug, Switzerland) was used for genomic DNase digestion. One microgram of the total RNA was reverse transcribed in a 25 *μ*L reaction volume with the Moloney murine leukemia virus (MMLV) reverse transcriptase (Promega, Dübendorf, Switzerland) and random primers. The resulting cDNA was diluted 1 : 20 and the absence of genomic DNA was confirmed with a reverse transcriptase control.

The quantitative real time polymerase chain reaction (qPCR) was performed with the SYBR green Fast Advance Master Mix (Qiagen) and a Rotor-Gene RG 2000 (Corbett Research, NSW, Australia), under the following conditions, 95°C for 5 min, followed by 40 cycles of 95°C for 5 second, and 60°C for 10 seconds. Specificity of the reaction was confirmed via melt curve analysis and the product size was confirmed on a 4% agarose gel.

The Genbank accession number and the primer sequences for all genes examined by qPCR are shown in Table 1.

### 2.3. Statistical Analysis

The most stable reference genes and the optimal combination to provide minimal variability were selected via the geNORM software program and a geometric mean of the four reference genes selected was used to normalise the expression of the genes of interest for both the eutopic and ectopic tissue [20]. The reaction efficiency of each assay was determined via linear regression [21] and the fold change calculated with the qBASEplus software (Biogazelle, Zwijnaarde, Belgium).

The difference between the matched eutopic and ectopic mRNA expression at different locations and the difference between mRNA in different ectopic locations were determined by a one-way Analysis of Variance (ANOVA) test with a *post hoc* Bonferroni's multiple comparisons test between selected groups. All values are presented as mean ± SEM and all statistical analysis was performed with Graphpad Prism 5.0 and significance was set at a value of *P* < 0.05.

## 3. Results

### 3.1. Cytokine mRNA Concentrations in Matching Eutopic and Ectopic Endometrial Tissue

**Figure 1:**
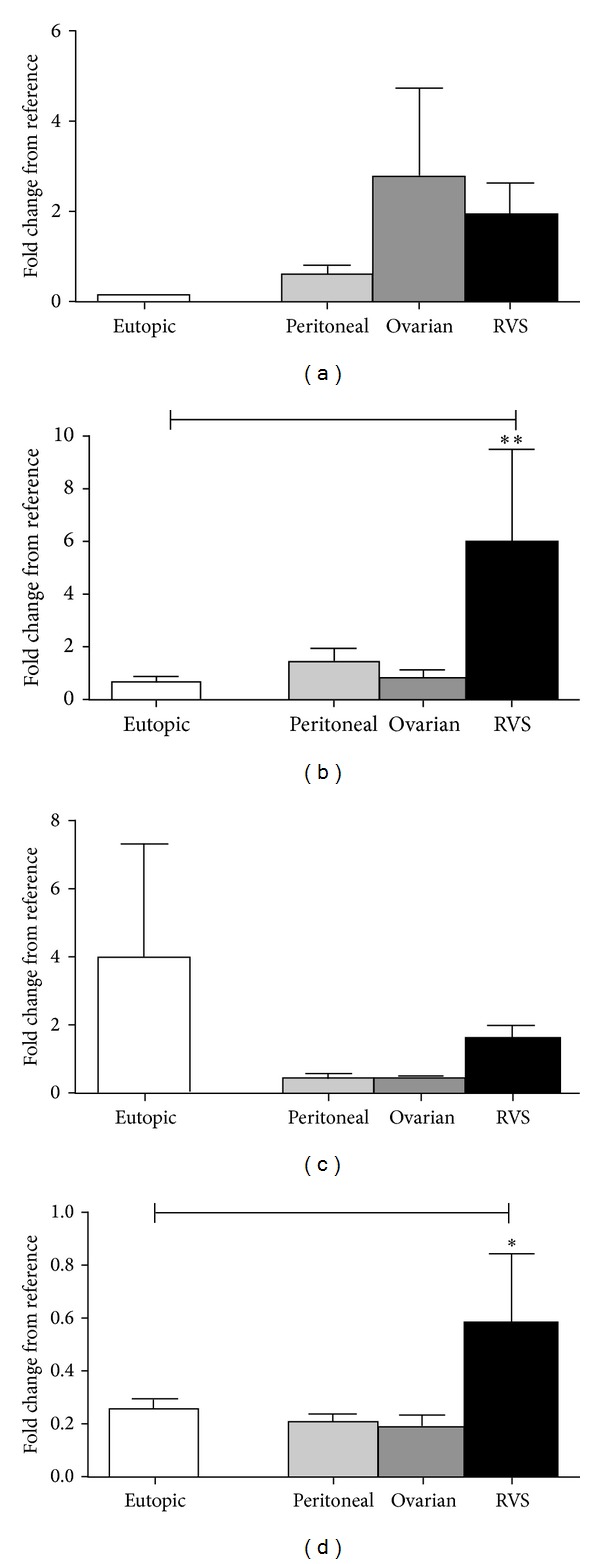
Chemokine production in eutopic endometrium and matching ectopic endometriotic lesions from different locations. (a) No significant difference was observed in the mRNA expression of MCP-1. (b) ENA-78 mRNA expression was significantly stronger in the RVS lesions compared to its matching eutopic tissue. (c) No significant difference in IL-8 mRNA expression was observed between the eutopic endometrium and the ectopic lesions from different locations. (d) RANTES expression was significantly higher in the RVS lesions compared to the eutopic endometrium. All values are represented by mean ± SEM. * < .05, ** < .01.

For the chemokines a one-way ANOVA test confirmed a significant variation between the mRNA concentrations of the ectopic endometriotic tissue with eutopic endometrial tissue for ENA-78 (*P* = 0.0039) and RANTES (*P* = 0.0490), but not for MCP-1 (*P* = 0.1251) or IL-8 (*P* = 0.7991) (Figure 1). A Bonferroni's multiple comparisons test was performed to compare the mean of each location against the eutopic mean. No significant difference was observed for MCP-1 mRNA expression between the eutopic tissue (0.107 ± 0.015, *n* = 17) and the ovarian lesions (2.751 ± 1.943, *n* = 8, *P* < 0.05), the peritoneal (0.590 ± 0.167, *n* = 7, *P* < 0.01) or the RVS (1.865 ± 0.712, *n* = 4, *P* < 0.01) lesions (Figure 1(a)). For ENA-78 there was a significantly stronger expression in the RVS lesions (5.905 ± 3.569, *n* = 4, *P* < 0.01) compared to the eutopic tissue (0.613 ± 0.250, *n* = 17), but no difference was observed in lesions from either the ovaries (0.811 ± 0.290, *n* = 8), or the peritoneum (1.444 ± 0.504, *n* = 7) (Figure 1(b)). For IL-8 there was no significant variation in the mRNA expression in either the peritoneum (0.396 ± 0.114, *n* = 8), the ovarian (0.409 ± 0.084, *n* = 8), or the RVS (1.574 ± 0.385, *n* = 5) compared to the eutopic tissue (3.979 ± 3.337, *n* = 20) (Figure 1(c)). A significantly higher expression of RANTES mRNA was observed in the RVS (0.582 ± 0.264, *n* = 5, *P* < 0.05) compared to the eutopic tissue (0.239 ± 0.0432, *n* = 17), but not in either the peritoneum (0.220 ± 0.030, *n* = 5) or the ovarian tissue (0.190 ± 0.045, *n* = 8) (Figure 1(d)).

**Figure 2:**
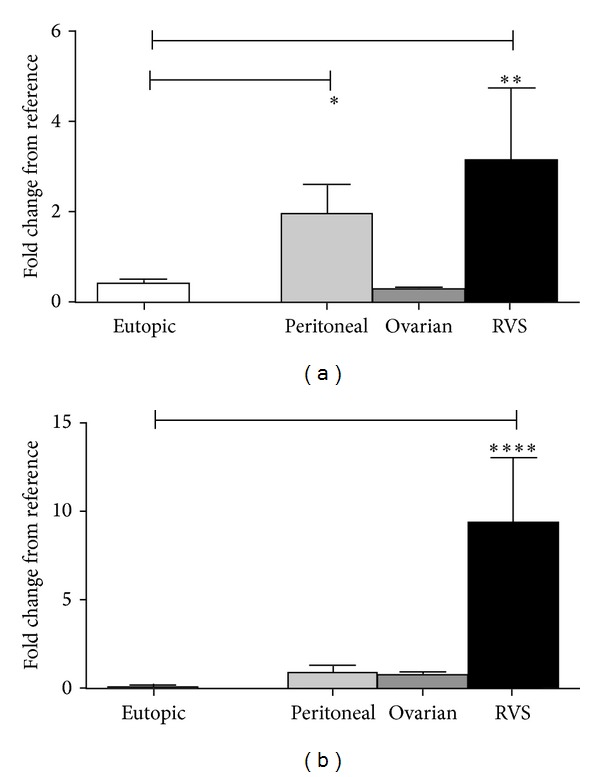
Inflammatory cytokine mRNA expression in eutopic and matching ectopic endometriotic lesions from different locations. (a) The mRNA expression of TNF*α* was significantly higher in the peritoneal and RVS lesions compared to their matching eutopic endometrium. (b) The mRNA expression of IL-6 was significantly higher only in the RVS lesions. All values are represented by mean ± SEM. * < .05, ** < .01, **** < .0001.

For the inflammatory cytokines a one-way ANOVA test confirmed a significant variation between the mRNA concentrations in the eutopic tissue with the mRNA concentration in the ectopic tissue for TNF*α* (*P* = 0.0014) and IL-6 (*P* < 0.0001) (Figure 2). A *post hoc* Bonferroni's multiple comparisons test indicated that TNF*α* mRNA expression in both the peritoneal (1.939 ± 0.667, *n* = 8, *P* < 0.05) and the RVS (3.128 ± 1.608, *n* = 4, *P* < 0.01) samples was significantly higher than that observed for their matching eutopic tissue (0.444 ± 0.106, *n* = 17), although no difference was observed with the ovarian lesions (0.291 ± 0.034, *n* = 8) (Figure 2(a)). For IL-6 there was a significantly higher expression in the RVS region (9.308 ± 3.714, *n* = 5, *P* < 0.0001), but not the ovaries (0.689 ± 0.237, *n* = 7) or the peritoneal region (0.667 ± 0.237, *n* = 7) compared to the eutopic tissue (0.152 ± 0.091, *n* = 17) (Figure 2(b)).

### 3.2. Cytokine mRNA Concentrations of Ectopic Endometriotic Lesions from Different Locations

**Figure 3:**
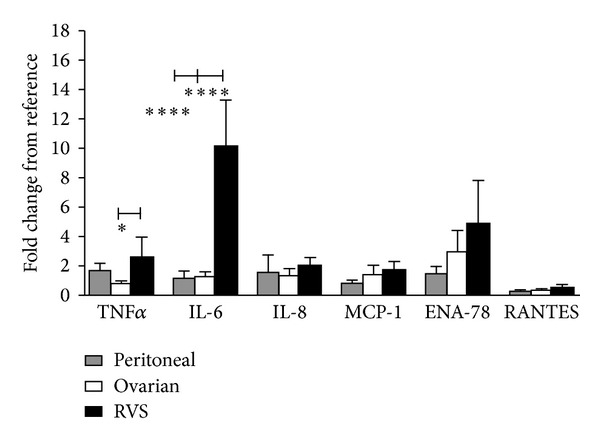
Comparison of cytokine and chemokine concentrations in endometriotic lesions from different locations. A comparison of the mRNA expression between endometriotic lesions from different locations indicated that TNF*α* expression in the RVS was significantly higher than expression in the ovarian lesions. IL-6 mRNA expression was significantly higher in the RVS than either the ovarian or the peritoneal lesions. There was no significant difference between the lesions with any of the other cytokines. All values are represented by mean ± SEM. * < .05, **** < .0001.

A significant variation was observed between the mRNA expression of TNF*α* (*P* = 0.0265) and IL-6 (*P* < 0.0001), amongst the endometriotic lesions from different locations. A *post-hoc* Bonferroni's multiple comparisons test indicated that the TNF*α* mRNA expression in the RVS (2.590 ± 1.357, *n* = 5) was significantly higher than in the ovarian lesions (0.813 ± 0.144, *n* = 24, *P* < 0.05), but not in the peritoneal lesions (1.711 ± 0.460, *n* = 12). For IL-6 the mRNA expression in the RVS lesions (10.150 ± 3.148, *n* = 6) was significantly higher than the expression in both the ovaries (1.260 ± 0.323, *n* = 24, *P* < 0.0001) and the peritoneum (1.211 ± 0.400, *n* = 13, *P* < 0.0001) (Figure 3).

In contrast no significant difference in mRNA expression was observed for any of the four chemokines examined in this study. MCP-1 expression in the RVS (1.700 ± 0.576, *n* = 5) was not significantly higher than either the ovarian (1.393 ± 0.632, *n* = 25) or the peritoneal samples (0.814 ± 0.215, *n* = 13), which was also the case for ENA-78 (Peritoneal; 1.497 ± 0.465, *n* = 13, ovarian; 2.988 ± 1.429, *n* = 25, RVS; 4.822 ± 2.969, *n* = 5), IL-8 (peritoneum; 1.548 ± 1.188, *n* = 13, ovaries; 1.352 ± 0.471, *n* = 25, RVS; 2.017 ± 0.543, *n* = 6), and RANTES (peritoneal; 0.288 ± 0.064, *n* = 11, ovarian; 0.364 ± 0.054, *n* = 22, RVS; 0.528 ± 0.222, *n* = 6) (Figure 3).

## 4. Discussion

The study showed that the mRNA expression of the chemokines ENA-78 and RANTES, as well as the inflammatory cytokines TNF*α* and IL-6, was significantly increased in the ectopic lesion compared to those in the matched eutopic tissue in women with endometriosis. For IL-6, ENA-78, and RANTES this increase was most significant in the RVS region, whereas for TNF*α*, it was in both the peritoneal lesions and the RVS lesions. In addition, when compared across lesion locations IL-6 was the most highly expressed in the RVS region compared to either the ovaries or the peritoneum. The results suggest therefore that different inflammatory proteins have separate roles in different lesions and understanding these roles may help to specifically target certain presentations of endometriosis. In addition, the increased production of many of these proteins by the RVS lesions provides some molecular evidence towards the notion that lesions developing in the RVS are strongly inflammatory.

Increased expression of chemokines by ectopic endometrial implants is an important early stage in the pathogenesis of endometriosis. Chemokines secreted by the ectopic lesions stimulate the infiltration of macrophages that further contribute to the development of the disease. In this study we found a significant increase in the expression of RANTES and ENA-78 in the RVS lesions compared to the matching eutopic tissue. RANTES production by ectopic lesions recruits leukocytes [22], which then in turn stimulates RANTES production [23] creating a feedback loop. Previous studies support this result as RANTES correlates with deep infiltrating endometriosis (DIE), which is most commonly found in the RVS [24]. ENA-78 may also play a significant role in the pathogenesis of endometriosis via the activation of macrophages and the adhesion of endometriotic cells to the underlying tissue [25]. Previous studies have shown that both endometrial epithelial [26] and stromal cells [27] produce significant amounts of ENA-78, which is stimulated by IL-1*β*, although this is the first evidence to indicate a significant upregulation in production of ENA-78 by RVS lesions.

IL-8 has strong chemotactic properties for neutrophils and T lymphocytes and is a potent angiogenic agent [28]. While numerous studies have shown an upregulation of IL-8 in the peritoneal fluid of women with endometriosis [29, 30] the source is not clear. An increase in peritoneal macrophages may be responsible for a higher concentration of IL-8, as would an increased production of IL-8 by the endometriotic lesions themselves.. Previous evidence shows that both cultured epithelial and stromal endometrial and endometriotic cells produce IL-8 [26, 27], although one study found that the ectopic tissue produced less IL-8 than the eutopic tissue [31]. Another study on cultured epithelial and stromal cells showed that IL-8 secretion is increased after exposure to IL-1*β* [26]. The lack of a significant difference for IL-8 in this study may be a reflection of the need to stimulate IL-8 production in endometriotic tissue.

TNF*α* mRNA expression was also significantly up-regulated in both the RVS and the peritoneal lesions compared to those in their matching eutopic tissue. For IL-6 a significant variation was only observed in the RVS lesion. TNF*α* has an essential role in the inflammatory process. The primary function of TNF*α* is to initiate a cascade of other cytokines that can further stimulate a proinflammatory response. In endometriosis it correlates with both the stage of the disease [32], and the menstrual pain reported [33]. Consistent with its early role in the inflammatory cycle it also stimulates cytokines, such as IL-6. The increased expression of TNF*α* is consistent with an important role for this cytokine in the early pathogenesis of endometriosis that may be common for different types of lesions. The fact that IL-6 is only significantly higher in RVS lesions may suggest that the inflammatory pathway between these two lesions may diverge prior to this point.

IL-6 is a multifunctional cytokine that can stimulate cell proliferation [34] and angiogenesis [35] and is hormonally regulated [36]. Different studies have shown both an increase [14, 15] or no change in the peritoneal fluid of women with endometriosis compared to women without [37]. IL-6 production has previously been identified in ectopic lesions, however results differ as to whether there is a change in production once the tissue becomes pathological. Some studies have found no significant difference between eutopic endometrium and endometriotic tissue from ovarian endometriosis [31], whereas others with ovarian endometriosis only [38], or non-detailed locations, have shown significant increases [39]. Furthermore, an *in vitro* study from endometrial stromal cells isolated from chocolate ovarian cysts showed a significant ability to produce IL-6 with production comparable to that of peritoneal macrophages [40]. None of these previous studies however have addressed the production of IL-6 in lesions from different locations.

A limitation of this study that should be mentioned is the small number of samples available for the RVS region. This is primarily due to the strict exclusion criteria for this study. As evidence indicates that the use of GnRHa can have an effect on the cytokine concentrations in the peritoneal fluid [41, 42] we excluded all samples from women with previous GnRHa use in the last 3 months. As women with RVS lesions are more likely to experience painful symptoms and to have previously sought treatment for endometriosis, a large proportion of women presenting to our tertiary care facility with RVS lesions had previous GnRHa or contraceptive use and thus were excluded from the study. However, although we only had a small number of samples, the ability to use matched eutopic and ectopic samples and our strict exclusion criteria should provide more weight to these results. Further studies with more samples should be performed to confirm our findings of differential cytokine production from lesions from different locations.

In conclusion, this study gives new insights in the production of chemokines and cytokines in endometriotic lesions from different locations and our results support the supposition that the RVS lesions are an intensely inflammatory form of endometriotic lesions. Assessing lesions from different locations uniquely may be vital in understanding the pathological changes of the disease and potentially for their mode of treatment.

## Figures and Tables

**Table 1:** Primer sequences of the reference genes and genes of interest.

Cytokine	Genbank accession no.	Sense	Antisense
GAPDH	NM_002046	5′-*TGC ACC ACC AAC TGC TTA GC*-3′	5′-*GGC ATG GAC TGT GGT CAT GAG*-3′
ACTB	NM_001101	5′-*CTG GAA CGG TGA AGG GTG ACA-*3′	5′-*AAG GGA CTT CCT GTA ACA ATG CA-3*′
YWHAZ	NM_003406	5′- *ACT TTT GGT ACA TTG TGG CTT CAA* -3′	5′-*CGC CAG GAC AAA CCA GTA T*-3′
RPL13A	NM_012423	5′*CCT GGA GGA GAA GAG GAA AGA-3*′	5′-*TTG AGG ACC TCT GTG TAT TTG TCA A-3*′
IL-6	NM_00600	5′-*GCA CTG GCA GAA AAC AAC CT*-3′	5′-*CAG GGG TGG TTA TTG CAT CT*-3′
IL-8	NM_000584	5′-*ACT GAG AGT GAT TGA GAG TGG AC*-3′	5′-*AAC CCT CTG CAC CCA GTT TTC* -3′
ENA-78	NM_02994	5′-*CTC CAA TCT TCG CTC CTC CAA*-3′	5′-*GGA GGC TCA TAG TGG TCA AGA G*-3′
TNF*α*	NM_000594	5′-*GCC CAT GTT GTA GCA AAC CC*-3′	5′-*TAT CTC TCA GCT CCA CGC CA-3*′
MCP-1	NM_002982	5′-*GGG CAT TGA TTG CAT CTG GC*-3′	5′-*CTG CTC ATA GCA GCC ACC TT-3*′
PAPP-A	NM_002581	5′-*AGT GGT ATC CTC ACC CTG CT-3*′	5′-*GTT GCA AAA GGC TCG GTT GT*-3′
RANTES	NM_002985	5′-*CTG CTT TGC CTA TGC CC*-3′	5′-*TCG GGT GAC AAA GAC GAC TG*-3′
